# Characterization of Cell Subpopulations Expressing Progenitor Cell Markers in Porcine Cardiac Valves

**DOI:** 10.1371/journal.pone.0069667

**Published:** 2013-07-23

**Authors:** Huan Wang, Balaji Sridhar, Leslie A. Leinwand, Kristi S. Anseth

**Affiliations:** 1 Department of Molecular, Cellular and Developmental Biology, University of Colorado, Boulder, Colorado, United States of America; 2 BioFrontiers Institute, University of Colorado, Boulder, Colorado, United States of America; 3 Department of Chemical and Biological Engineering, University of Colorado, Boulder, Colorado, United States of America; 4 Howard Hughes Medical Institute, University of Colorado, Boulder, Colorado, United States of America; Brigham and Women's Hospital, Harvard Medical School, United States of America

## Abstract

Valvular interstitial cells (VICs) are the main population of cells found in cardiac valves. These resident fibroblastic cells play important roles in maintaining proper valve function, and their dysregulation has been linked to disease progression in humans. Despite the critical functions of VICs, their cellular composition is still not well defined for humans and other mammals. Given the limited availability of healthy human valves and the similarity in valve structure and function between humans and pigs, we characterized porcine VICs (pVICs) based on expression of cell surface proteins and sorted a specific subpopulation of pVICs to study its functions. We found that small percentages of pVICs express the progenitor cell markers ABCG2 (~5%), NG2 (~5%) or SSEA-4 (~7%), whereas another subpopulation (~5%) expresses OB–CDH, a type of cadherin expressed by myofibroblasts or osteo-progenitors. pVICs isolated from either aortic or pulmonary valves express most of these protein markers at similar levels. Interestingly, OB–CDH, NG2 and SSEA-4 all label distinct valvular subpopulations relative to each other; however, NG2 and ABCG2 are co-expressed in the same cells. ABCG2^+^ cells were further characterized and found to deposit more calcified matrix than ABCG2^-^ cells upon osteogenic induction, suggesting that they may be involved in the development of osteogenic VICs during valve pathology. Cell profiling based on flow cytometry and functional studies with sorted primary cells provide not only new and quantitative information about the cellular composition of porcine cardiac valves, but also contribute to our understanding of how a subpopulation of valvular cells (ABCG2^+^ cells) may participate in tissue repair and disease progression.

## Introduction

Human cardiac valves open and close over 100,000 times a day ensuring directional flow of blood in the heart [1]. The cyclic movement and mechanical stress of valves require that the tissue has the capacity to repair damage that may occur during normal function. This remodeling is thought to be mediated by the main cell population found in the valve, valvular interstitial cells (VICs), since these cells have reversible and dynamic phenotypes and build the matrix structure in prenatal and postnatal valves [2–4]. VICs play critical functions in maintaining valve homeostasis through secreting not only extracellular matrix components (e.g., collagen and fibronectin), but also matrix remodeling enzymes, such as matrix metalloproteases (MMPs) [5,6]. Normal aortic valves are comprised of three distinct matrix layers, rich in elastin, proteoglycan and collagen, implying that VICs residing in these tissue sub-domains may have different fates or phenotypes [7]. In response to valvular diseases such as myxomatous valves, VICs have been shown to be activated to myofibroblasts, which produce excessive levels of collagen and MMPs [8]. In valve calcification, cells residing in the leaflets have been shown to adopt an osteoblast-like phenotype and actively mediate calcification of the valves [9,10]. Collectively, these data suggest that cellular fates and functions of VICs play critical roles in determining whether heart valves are in a healthy or a diseased state.

Despite the causal relationship between VICs and valve function, it is less clear how heterogeneous the cellular composition of valves is and how different subpopulations of VICs might differentially regulate valve homeostasis or disease progression. Latif et al. [9] reported that VICs from healthy human valves (hVICs) express the fibroblast markers fibroblast surface antigen (FSA) and vimentin, but that only a small fraction of hVICs express the human mesenchymal stem cell (hMSC) marker CD105 or the smooth muscle cell markers desmin and smooth muscle myosin based on immunocytochemistry. Bovine VICs (bVICs) have been shown to express integrin β1 (or CD29) and type A nonmuscle myosin, but not the hematopoietic marker CD45 nor the endothelial/epithelial marker von Willebrand Factor (vWF) [11]. Further, several pieces of evidence suggest that there are progenitor cells in VICs. First, isolated porcine VICs (pVICs) cultured *in vitro* have been shown to differentiate into three distinct lineages: osteoblasts, adipocytes and chondrocytes given appropriate chemical cues [12]. Second, ~0.5% of pVICs have the side population stem cell property of excluding the Hoechst 33342 DNA dye and to differentiate into myofibroblasts when cultured in high serum media [13]. Additionally, c-Kit^+^ progenitor cells have been observed in hVICs [14], and bone marrow-derived stem cells migrate to human and mouse cardiac valves and differentiate into fibroblast-like cells [15,16]. Therefore, VICs are heterogeneous, composed primarily of fibroblasts and small percentages of myofibroblasts, smooth muscle cells and progenitor cells [10]. Together, these cells must coordinately regulate valvular functions.

Since healthy human cardiac valves are of limited availability and porcine cardiac valves mimic human cardiac valves closely in structure and have been utilized as a surrogate tissue for human valve replacement, we sought to better characterize pVICs from porcine valve leaflets. To define the different subpopulations of pVICs in a quantitative manner, we utilized fluorescence activated cell sorting (FACS) to characterize cell surface protein expression and to sort specific pVIC subpopulations based on those markers to study their cellular functions. A collection of cell surface protein markers (Table 1) was used to quantify the percentage of different subpopulations in porcine aortic and pulmonary VICs. These candidate markers were selected to serve as negative or positive selection markers based on their relevance to fibroblast biology. For example, CD31 primarily marks endothelial cells [17] and is not expected to be present on pVICs. OB-cadherin (OB–CDH) is a type of cadherin commonly expressed in mesenchymal cells, and expression of OB–CDH is significantly elevated during myofibroblast activation in skin granulation tissue [18]. We hypothesized that OB–CDH labels resident myofibroblasts in valves. Furthermore, to address the question of whether there exist progenitor cells in the VIC population, we also examined a number of progenitor cell markers identified for other tissues, including a side population progenitor cell marker, ABCG2 (ATP-binding cassette, sub-family G, member 2) [19], a pericyte marker, NG2 (or chondroitin sulfate proteoglycan 4) [20], and an embryonic and mesenchymal stem cell marker, SSEA4 (stage-specific embryonic antigen 4) [21,22]. The FACS-based method not only enables us to identify and quantify various pVIC subpopulations, but also makes it possible to reproducibly isolate desired subpopulations to study their molecular functions *in vitro*. Understanding the functions of distinct VIC subpopulations in aortic valves may help direct new therapeutic treatments targeting potentially pathogenic subpopulations. Additionally, this technique may also allow isolation and transplantation of potential progenitor cells into diseased valves as a treatment.

**Table 1:** Cell Surface Protein Markers.

**Marker Name**	**Cell type(s) marked**	**References**
CD31	Endothelial cells	[17]
OB–CDH	Myofibroblasts, Mesenchymal cells	[18]
ABCG2	Side population progenitor cells	[19,51]
NG2	Pericytes, Progenitor cells	[20,39]
SSEA4	Human embryonic stem cells, hMSCs	[21,22]

## Materials and Methods

### 2.1: Cell Isolation and Culture

Porcine hearts were obtained from Hormel Foods Corporation (Austin, MN, USA) within 24 hours of sacrifice. Primary pVICs were harvested from porcine aortic or pulmonary valve leaflets based on a sequential collagenase digestion as described previously [23]. Specifically, the aortic or the pulmonary valves were excised from the pig hearts and put into wash buffer comprised of Earle’s Balanced Salt solution (Sigma, Cat# E2888), 50U/ml penicillin, 50µg/ml streptomycin, and 0.5µg/ml fungizone. After the wash, the leaflets were incubated in 250U/ml Collagenase Type II solution (Worthington Biochemical Corporation, Cat# LS004176, prepared in the wash buffer) for 30min at 37°C. The samples were then vortexed at a maximum speed for 30 seconds to remove endothelial cells. The leaflets were washed one time and then subjected to a second round of digestion with the Collagenase Type II solution for 1 hour and vortexed for 2 minutes to collect pVICs. The isolated pVICs were sorted and grown in growth media [advanced DMEM/F12 (1:1) (Life technologies, Cat# 12634010) or Medium 199 (Life technologies, Cat# 11150067) with 15% Fetal Bovine Serum (FBS), 50U/ml penicillin, 50µg/ml streptomycin, and 0.5µg/ml fungizone]. Media was refreshed every 2-3 days. To characterize the cell recovery (Figure S1), the leftover tissue from the second collagenase digestion was subjected to another collagenase digestion for 20 hours at 37°C, which completely digested the tissue. The total cell number was then counted at each digestion step to quantify the percentage of pVICs released.

### 2.2: Flow Cytometry

Freshly isolated aortic or pulmonary pVICs were strained through 100µm sieves twice and washed with cold PBS once. The cells were subsequently stained with selected primary antibodies raised against ABCG2 (R&D Systems, Cat # FAB995P), CD31 (AbD Serotec, Cat# MCA1746APC), OB–CDH (clone 15F7 [24], a generous gift from Dr. Micheal Brenner’s group at the Brigham and Women’s Hospital) and SSEA4 (R&D Systems, Cat# FAB1435P) for 1 hour at 4°C. Samples were washed once with cold PBS after staining. 0.1ng/μl DAPI was added to all samples to distinguish dead cells during flow cytometry. The percentage of cells expressing these cell surface markers was quantified on a CyAN ADP flow cytometer (Dako); over 10,000 events were collected for each sample and 3 or more biological replicates were profiled for each marker or co-staining. Cells were subsequently sorted based on positive or negative staining for ABCG2, gated with low DAPI staining (live cells) through fluorescence activated cell sorting (FACS) on a MoFlo high-speed cell sorter (Dako). 

### 2.3: Cryosection of valve leaflets

After isolation, pulmonary or aortic valve leaflets were placed in cryomolds with O.C.T. medium (Tissue-Tek) to be frozen. Freezing was performed by placing the mold in isopentane that was cooled by liquid nitrogen. After solidification, cryomolds were removed from the isopentane, dried with Kimwipes, and stored at -80^°^C until cryosectioning. Frozen blocks were cryosectioned using a Cryostat (Leica) at -21^°^C. For each sample block, 15-µm longitudinal cross-sections of valves were collected on SuperFrost Plus Gold slides (Fisher Scientific). The slides were stored at -20^°^C until immunohistochemistry.

### 2.4: Immunohistochemistry

After cryosectioning, sections were ﬁxed in 10% formalin for 20 min at room temperature. Sections were analyzed by anti-CD31 (Genway 1:100), anti-NG2 (Chemicon, 1:200), anti-OB–CDH (15F7 clone, 1:100), and anti-SSEA-4 (R&D systems, 1:10). Sections were digested using Collagenase II (Worthington) at 250 U/ml at 37^°^C for 15 minutes to expose the antigens. For anti-NG2 staining, boiled Retrievagen (BD Pharmigen, pH 6) was applied for 30 min at 60^°^C. After primary antibody incubation and washes, sections were subsequently probed with AlexaFluor 488 or 594-conjugated secondary antibodies and counterstained with Prolong Gold with DAPI (Life Sciences) for cell nuclei. A laser scanning confocal microscope (Zeiss, LSM710) was used to acquire images with a 20X water objective using the same settings and post-processing for all images. Negative controls were performed on sections that received no primary antibody treatment, showing no positive staining.

### 2.5: Osteogenic Differentiation

Sorted cells were expanded in growth media for ~2 weeks before re-seeding at ~300,000 cells/cm^2^ for differentiation. As a control, cells were cultured in control medium [High glucose DMEM (Life Technologies, Cat# 11965-092) supplemented with 10% FBS, 50U/ml penicillin, 50µg/ml streptomycin, and 0.5µg/ml fungizone]. For osteogenic differentiation, cells were treated with osteogenic medium [derived from the control medium supplemented with 100nM dexamethasone (Sigma, Cat# D1756), 50 μM ascorbic acid 2-phosphate (Sigma, Cat# 49752), and 20mM β-glycerophosphate (Sigma, Cat# G9891)] for 8 days. Media was changed every 2-3 days.

### 2.6: Calcium Deposition Assay

Calcium deposition was assayed using a Calcium Reagent Set (Pointe Scientific Inc), as described previously [25]. Briefly, the matrix deposited by cells cultured in osteogenic media was solubilized with 0.6N HCl overnight at 4°C. The supernatant was collected and diluted appropriately before mixing with the calcium reagent solution at 1:1. Absorbance was measured at 560nm. The relative calcium deposition was calculated by normalizing to the ABCG2^-^ cells under the osteogenic condition at day 8.

### 2.7: Statistics

One-way ANOVA was used to compare data sets and a *p* value less than 0.05 was considered statistically significant.

## Results

### 3.1: Freshly isolated aortic pVICs express distinct cell surface markers

**Figure 1:**
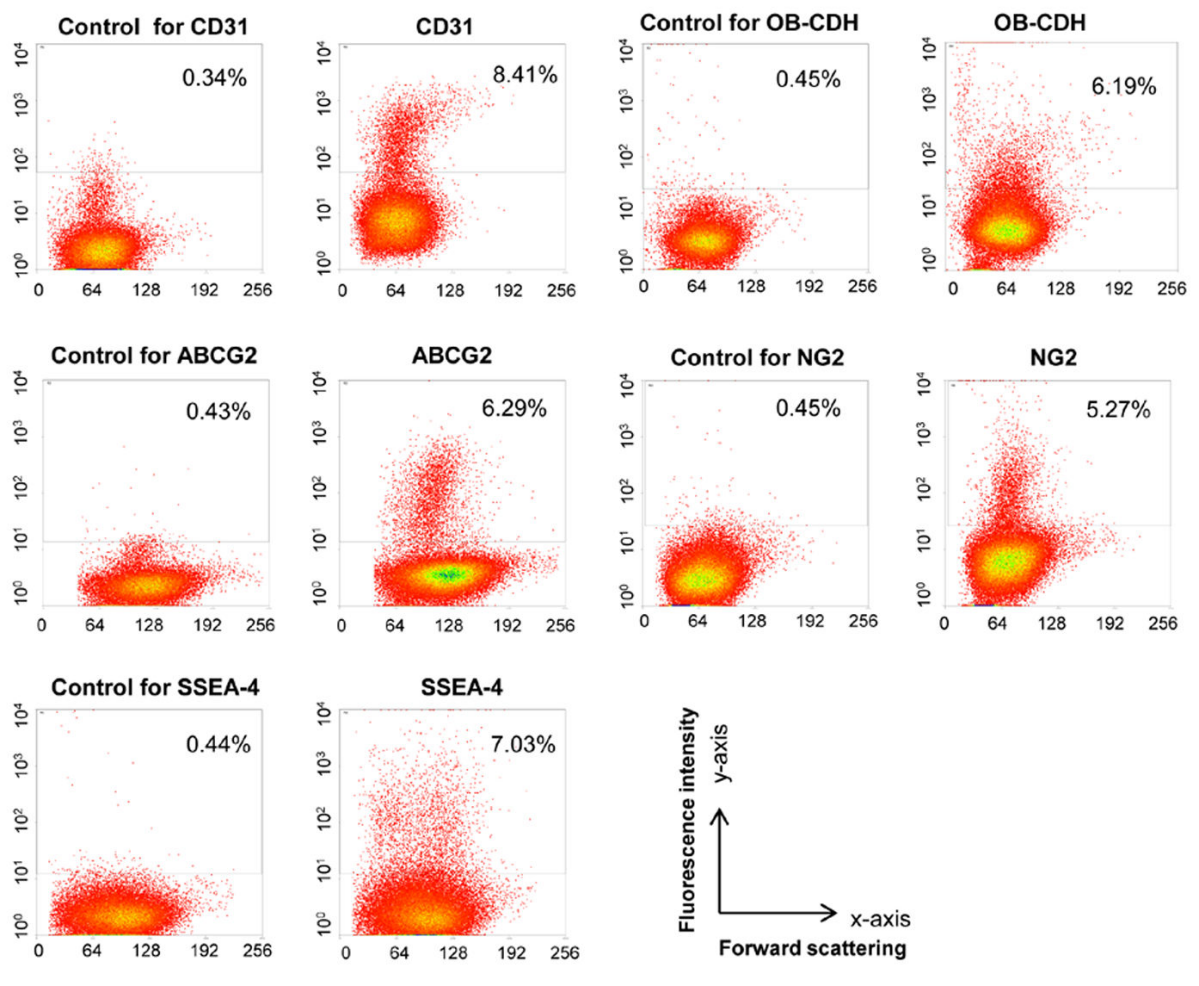
Freshly isolated aortic pVICs express distinct cell surface markers. Freshly isolated aortic pVICs were stained with antibodies for CD31, OB–CDH, ABCG2, NG2, SSEA-4, and the corresponding control antibodies. Staining was quantified by flow cytometry. In the figure, the y-axis is fluorescence intensity and the x-axis is forward scattering. Percentage in the rectangular gates represents the fraction of positively stained cells. After subtracting the background, about 7.70% of these aortic pVICs stained positive for CD31, 4.71% stained positive for OB–CDH, 5.60% stained positive for ABCG2, 5.56% stained positive for NG2, and 6.59% stained positive for SSEA-4.

pVICs, the main cell population in porcine cardiac valves, play an important role in mediating tissue homeostasis and repair. However, it is still not clear how many different subpopulations are present in VICs and whether or not these different populations have distinct molecular and cellular functions. To better define the heterogeneity of pVICs, primary pVICs were isolated from porcine aortic valves by a collagenase digestion, which releases close to 80% of pVICs from the tissue (Figure S1). The primary cells were subsequently stained with antibodies recognizing endothelial cells (CD31), certain mesenchymal cells (OB–CDH), or some progenitor cells (ABCG2, NG2 and SSEA-4). As shown in Figure 1, less than 10% of aortic pVICs express CD31, OB–CDH, ABCG2, NG2 and SSEA-4. Quantification of the cell marker expression is summarized in Table 2 for pVICs isolated from both aortic and pulmonary valves.

**Table 2:** Unique cell subpopulations identified based on cell surface markers are present in both aortic and pulmonary valves.

**Cell surface marker**	**Aortic pVICs**	**Pulmonary pVICs**
CD31	8.24 + 0.13%	7.38 + 0.61%
OB–CDH	5.07 + 0.93%	5.66 + 1.46%
ABCG2	5.52 + 0.90%	5.97 + 1.15%
NG2	5.26 + 0.49%	4.50 + 0.63%
SSEA4	6.93+ 0.89%	14.42 + 0.41%*

The percent of pVICs expressing specific cell surface markers was quantified by flow cytometry. Data are presented as the average percentage + standard error. * indicates significant difference between aortic and pulmonary pVICs (p<0.05).

### 3.2: Aortic and pulmonary pVICs express similar levels of the cell surface markers examined

To compare cellular composition of pVICs isolated from different types of cardiac valves, we characterized pVICs isolated from both aortic and pulmonary valves by flow cytometry based on the cell surface markers listed in Table 1. We found that pVICs expressed similar levels of most of the cell surface markers independent of the valve type. As presented in Table 2, 8.24 + 0.13% of aortic pVICs and 7.38 + 0.61% of pulmonary pVICs express CD31, which are probably derived from valvular endothelial cells during isolation. In addition, 5.07 + 0.93% of aortic pVICs and 5.66 + 1.46% of pulmonary pVICs express OB–CDH. We also examined three markers associated with progenitor cells (ABCG2, NG2 and SSEA-4), and found that pVICs from both types of valves expressed similar levels of ABCG2 (5.52 + 0.90% for aortic pVICs and 5.97 + 1.15% for pulmonary pVICs) and NG2 (5.26 + 0.49% for aortic pVICs and 4.50 + 0.63% for pulmonary pVICs). However, significantly more pVICs from pulmonary valves expressed SSEA-4(14.42 + 0.41%) than those from aortic valves (6.93+ 0.89%). While these data provide quantification of different subpopulations based on the presence of single protein markers, it is not clear whether these different subpopulations are distinct, or whether they are from the valvular endothelium.

### 3.3: OB–CDH, NG2 and SSEA-4 define distinct valvular subpopulations, while NG2 and ABCG2 are co-expressed by the same cell subpopulation

**Figure 2:**
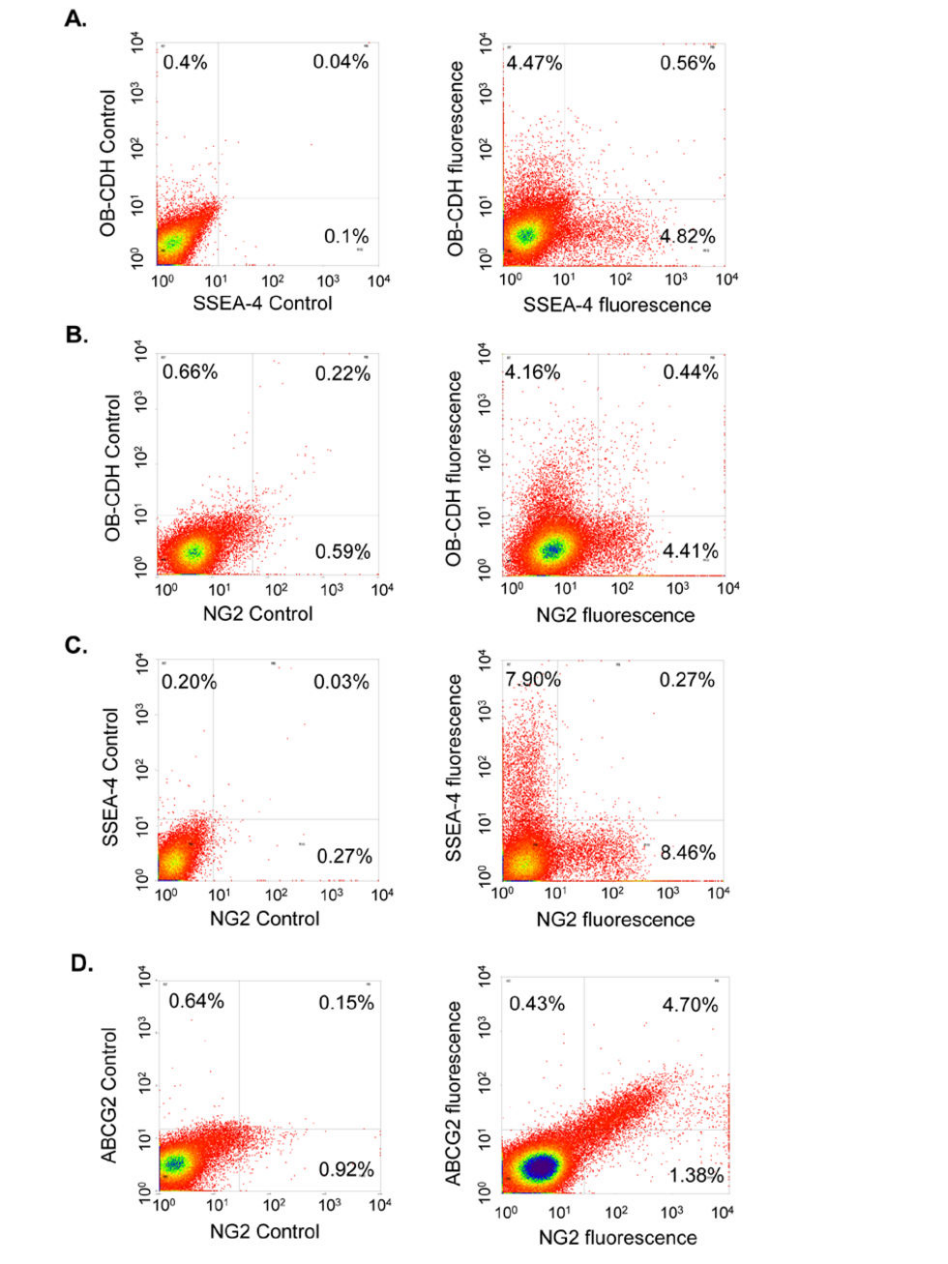
OB–CDH, NG2 and SEA-4 define distinct valvular subpopulations, but NG2 and ABCG2 are co-expressed by the same cell subpopulation. To examine co-expression of these different markers, freshly isolated pVICs were co-stained with pairwise combinations of the markers OB–CDH, NG2 and SSEA-4, or with ABCG2 and NG2. Both the x-axes and the y-axes are fluorescence intensity of the antibody staining. (A) OB–CDH was not co-expressed with SSEA-4 in the same cell population. (B) OB–CDH was not co-expressed with NG2 in the same cell population. (C) SSEA-4 was not co-expressed with NG2 in the same cell population. These data support that these markers (OB–CDH, NG2 and SSEA-4) label distinct subpopulations relative to each other in pVICs. (D) However, NG2 and ABCG2 were co-expression by the same cell subpopulation in aortic valves.

To examine whether these different cell surface markers are co-expressed, we carried out double staining for pairs of cell surface markers. We found that OB–CDH^+^ pVICs did not express SSEA-4 or NG2 (Figure 2A and 2B). In addition, NG2^+^ pVICs were negative for SSEA-4 (Figure 2C). All the data support that these three protein markers (OB–CDH, NG2 and SSEA-4) label distinct pVIC subpopulations relative to each other. However, ABCG2 and NG2 were co-expressed by the same cell subpopulation (Figure 2D).

### 3.4: SSEA-4 staining does not overlap with CD31, whereas OB–CDH or ABCG2 staining partially overlaps with CD31

**Figure 3:**
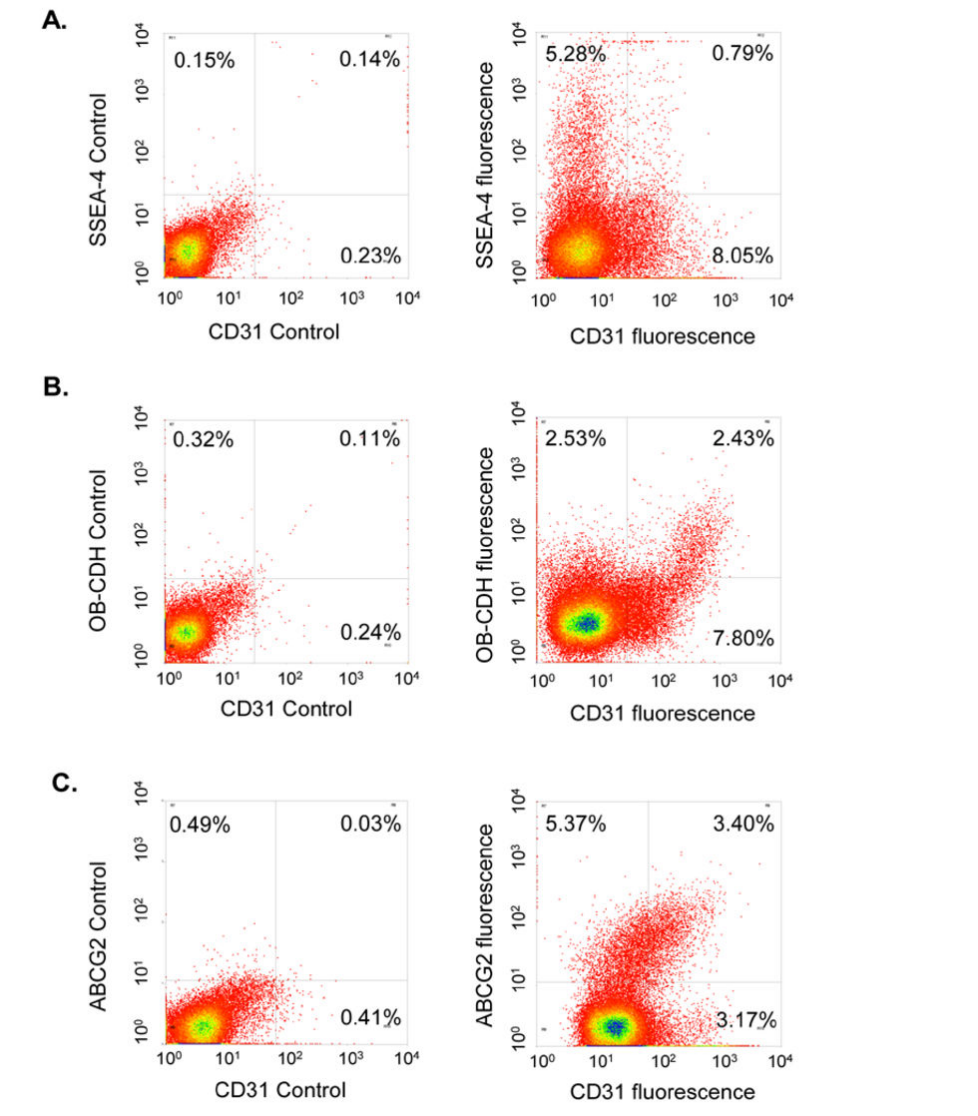
SSEA-4 staining does not overlap with CD31, whereas OB–CDH or ABCG2 staining partially overlaps with CD31. To examine the origin of these cell subpopulations, aortic pVICs were double stained with CD31 and one of the following markers: SSEA-4, OB–CDH or ABCG2. CD31 is expressed by porcine valvular endothelial cells but not by pVICs. From (A), SSEA4 and CD31 labeled distinct cell subpopulations. However, OB–CDH^+^ or ABCG2^+^ cells showed heterogeneous CD31 staining. About 52% of OB–CDH^+^ cells were CD31^-^ and ~61% of ABCG2^+^ cells were CD31^-^, indicating that a majority of these cells are from pVICs rather than endothelium.

We then asked whether these valvular cell subpopulations were derived from pVICs or endothelial cells. As shown in Figure 3A, SSEA4^+^ cells are mostly negative for CD31, an endothelial cell marker, and are therefore derived from pVICs. However, OB–CDH or ABCG2 staining partially overlaps with CD31 expression, suggesting that these cells are from both pVICs and endothelial cells.

### 3.5: Localization of certain pVIC subpopulations in native porcine valves

**Figure 4:**
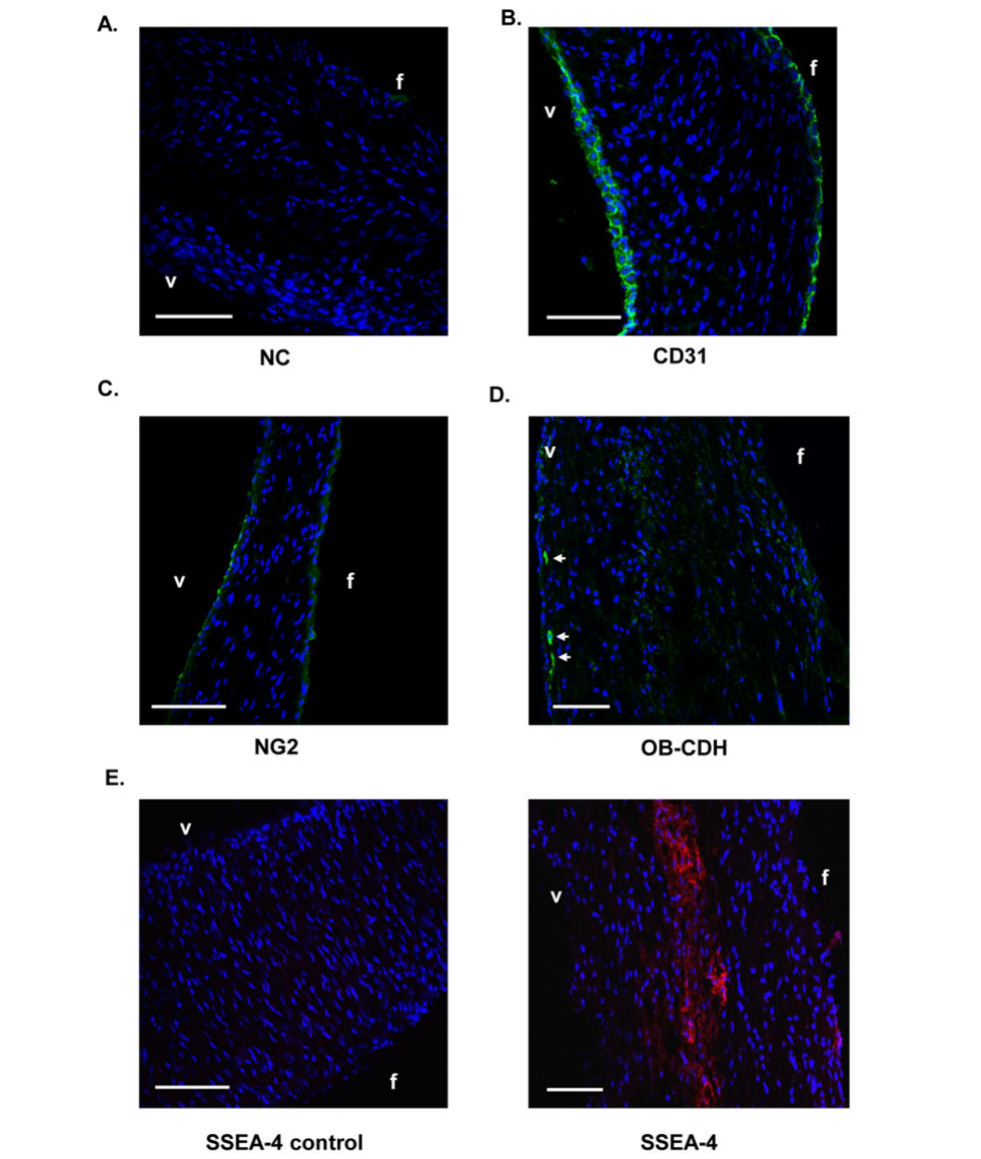
Localization of certain pVIC subpopulations in native porcine valves. Immunostaining of multiple VIC subpopulation markers was carried out on aortic valve sections. v: ventricularis side. f: fibrosa side. (A) Negative control (NC) which has been incubated with secondary antibody only and serves as a control for panels B–D. (B) CD31 staining (green) localized to the valve surfaces. Interestingly, some surface regions had multiple layers of CD31^+^ endothelial cells. (C) NG2 staining (green) was found at the edge of the valves. (D) OB–CDH stained clusters of pVICs on the ventricularis side of the aortic valves (green, arrows). (E) Compared with control, SSEA-4 staining (red) was primarily in the middle of the valves.

To examine the relative localization of valvular cells expressing different markers in valve leaflets, we stained aortic valve sections with the following markers: CD31, NG2, OB–CDH and SSEA-4. The image in Figure 4A is a negative control (NC) stained with the secondary antibody only. As expected, CD31 labeled endothelial cells around the valve section and sometimes labeled more than one layer of cells (green, Figure 4B). NG2 was found to label some cells along the edge of the valve (green, Figure 4C). OB–CDH stained a few clusters of pVICs close to the ventricularis (green, Figure 4D). However, SSEA-4 labeled pVICs residing near the middle of valves (red, Figure 4E).

### 3.6: ABCG2^+^ valvular cells deposit more calcified matrix than ABCG2^-^ cells in osteogenic culture conditions

**Figure 5:**
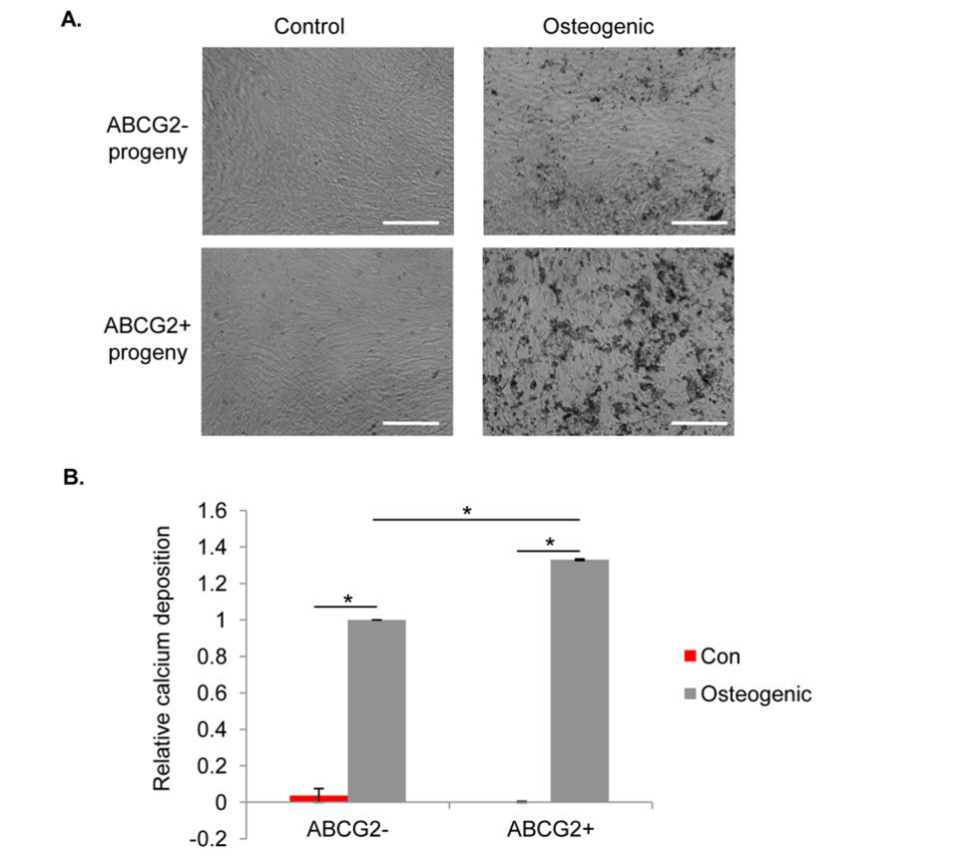
ABCG2^+^ valvular cells deposit more calcified matrix than ABCG2^-^ cells in osteogenic conditions. Sorted ABCG2^+^ and ABCG2^-^ valvular cells were grown to confluence and re-plated in osteogenic media for 8 days. (A) Brightfield images of cells cultured in control conditions or osteogenic conditions were taken at day 8. ABCG2^+^ progeny secreted more dark mineralization spots than ABCG2^-^ progeny on plastic plates. Scale bar: 100 µm. (B) Calcium deposition by the cells was quantified as described in the Materials and Methods. Data was represented as mean + standard error. Compared with ABCG2^-^ progeny, ABCG2^+^ progeny produced higher amounts of calcium composites at day 8 (* indicates p<0.05 in between the groups).

In order to study *in vitro* the molecular functions of cells expressing the SP stem cell marker ABCG2, we sorted similar numbers of ABCG2^+^ and ABCG2^-^ valvular cells by FACS. A majority of ABCG2^+^ and ABCG2^-^ cells showed a fibroblastic morphology in culture. We assessed the osteogenic differentiation potential of the sorted cells based on established chemical induction protocols [26]. As shown in Figure 5A, ABCG2^+^ progeny produced a larger area of mineralized matrix (dark spots) than ABCG2^-^ progeny in osteogenic culture conditions. The amount of calcium in the matrix secreted by the cells was quantified (Figure 5B), and ABCG2^+^ progeny deposited more calcium than ABCG2^-^ progeny after 8 days of culture in osteogenic media.

## Discussion

For many years, VICs have been primarily defined based on their location in valve leaflets, and researchers have isolated VICs and studied this collective cell population. In this study, we aim to better define the VIC population by characterizing its diversity based on the expression of five selected cell surface proteins (Table 1). Our results show that small fractions of freshly isolated pVICs are positive for: i) OB–CDH, a mesenchymal / myofibroblastic marker, ii) NG2, a pericyte progenitor marker, iii) SSEA-4, a stem cell marker, or iv) ABCG2, a SP stem cell marker. SSEA-4^+^ cells are mostly negative for CD31; however, OB–CDH or ABCG2 expression partially overlaps with CD31 expression. Intriguingly, ~5% of pVICs express both ABCG2 and NG2, two progenitor cell markers. About 60% of these cells come from pVICs, whereas the rest are derived from CD31^+^ valvular endothelial cells. As one of the main valvular diseases, calcific aortic stenosis, has been associated with an osteoblast phenotype of resident VICs [9], we sought to characterize the osteogenic potential of the ABCG2 subpopulations. After 8 days of culture under osteogenic conditions, the ABCG2^+^ cells deposited a more mineralized matrix than the ABCG2^-^ cells, as measured by calcium deposition. These results support the notion that ABCG2^+^ valvular cells may participate in the progression of valve calcification. The cell sorting-based method presented here may be useful in isolating, characterizing, and identifying cellular determinants in valvular diseases.

When pVICs are isolated from their native matrix and cultured on plastic plates, a majority of them adopt a similar fibroblastic morphology. However, differences at the molecular level cannot be easily distinguished based on morphology, and there is a growing appreciation of the diverse and plastic phenotypes of VICs [10]. For example, pVICs cultured on plastic plates are a heterogeneous population with both fibroblasts and myofibroblasts. Specific protein markers expressed by the cells, which can be recognized by their corresponding antibodies, have served as a useful tool to differentiate and to select specific cell types or cell subpopulations for further study. CD31, or platelet endothelial cell adhesion molecule 1 (PECAM-1), has been established as an endothelial cell marker and can be used to identify endothelial cells lining the valve [17,27,28]. As shown in Figure 1, ~8% of freshly isolated aortic pVICs express CD31, which is likely due to contamination with a small number of valvular endothelial cells during pVIC isolation. In traditional pVIC isolation, a sequential collagenase digestion is performed, where the first collagenase treatment is used to remove endothelial cells. However, based on our staining of CD31 on valve tissue sections (Figure 4B), CD31 primarily labels valvular endothelial cells located around the edges of valve sections, but sometimes there are multiple layers of CD31^+^ cells on valve surfaces. This could explain why the initial collagenase digestion might not have removed the endothelial layer completely from the valves. Consistent with immunocytochemistry data from Rattazi et al. [11], our flow cytometry and immunohistochemistry data (Figures 1 and 4B) show that a majority of pVICs in the leaflets do not express this endothelial lineage marker (CD31), supporting the mesenchymal origin of these cells.

As aortic valves and pulmonary valves are surgically more accessible than the other two types of cardiac valves (mitral and tricuspid valves), we compared pVICs isolated from aortic and pulmonary valves and showed that they had similar expression of many cell surface markers (Table 2). For example, similar percentages of pVICs from either aortic or pulmonary valves expressed CD31, OB–CDH, ABCG2, NG2, or SSEA-4 (Table 2). This result suggests that pVICs from aortic and pulmonary valves have a similar cellular composition based on these markers. We further evaluated the efficiency of the collagenase digestion and found that around 78.3 ± 7.9% of pVICs are released from the valves during the second collagenase digestion (Figure S1). This result suggests that we are characterizing the majority of the pVIC population from the valves. Meanwhile, the VIC isolation protocol used in this study has been widely accepted in the valve biology field to minimize cell death and cleavage of cell surface proteins induced by long-term collagenase treatment. Our analysis provides relevant quantification for the majority of pVICs, which are often used for *in vitro* cell culture studies.

Interestingly, 5-6% of pVICs from either the pulmonary or the aortic valve express OB–CDH (Table 2). OB–CDH expression has been associated with a pathogenic myofibroblast phenotype. During skin wound healing, resident fibroblasts are activated to myofibroblasts, which is accompanied by increased expression of OB–CDH and decreased expression of N-cadherin (N–CDH) [18]. The OB–CDH bonds can resist two fold higher forces than N–CDH junctions in fibrogenic fibroblasts, indicating that these cells increase their adhesion to surrounding cells during fibrogenesis [29]. Additionally, staining on porcine valve sections shows that clusters of OB–CDH^+^ pVICs localize to the ventricularis side of valves (Figure 4D), which mimics staining of another myofibroblast marker, α-smooth muscle actin. The OB–CDH^+^ pVICs may very well be resident myofibroblasts in porcine cardiac valves. As numerous research groups have sought to characterize the myofibroblast properties of pVICs in both valvular regeneration and pathogenesis [3,8,13,30–32], sorting and characterization of this OB–CDH^+^ subpopulation, especially as related to their functions in tissue remodeling and matrix deposition, warrant further investigation. OB–CDH has also been shown to be expressed by osteoblasts and directly regulates osteogenic differentiation [33–35], so its role in relation to the osteoblast phenotype of VICs during valve calcification could be studied further. It is unexpected that OB–CDH also labels some valvular endothelial cells (CD31^+^) based on flow cytometry (Figure 3B) because OB–CDH is mostly expressed by mesenchymal cells during development [36]. However, previous literature has not shown that endothelial cells do not express OB–CDH, and OB–CDH^+^ pVICs might be specifically sorted based on CD31^-^ cells in order to study their specific functions.

We have also defined several pVIC subpopulations expressing progenitor cell markers, including ABCG2 (5-6%), NG2 (~5%) and SSEA4 (7-14%), in both aortic and pulmonary valves (Table 2). Interestingly, significantly more SSEA-4^+^ pVICs are identified in pulmonary valves than in aortic valves (Table 2). SSEA-4 is a marker for embryonic stem cells and hMSCs [21,22] and may mark specific progenitor subpopulations in the valve. Latif et al. found that <10% of hVICs expressed the hMSC maker CD105 based on immunocytochemistry [5]. Consistently, we have observed that 7-14% of pVICs express a different hMSC marker, SSEA-4, based on flow cytometry. It will be worthwhile to sort these pVICs and study their similarities to MSCs, especially their potential fate during valvular repair and disease progression. Additionally, the different levels of SSEA-4 expression in pulmonary valves vs. aortic valves could be related to the structural differences of these valves and their regenerative capacity in response to mechanical insults or diseases. With further characterization, we have found that these SSEA-4^+^ cells are almost exclusively derived from pVICs, since they are negative for CD31 staining (Figure 3A). Our immunostaining data on aortic valve sections is consistent with this in showing that SSEA-4 staining is localized to the middle of the leaflets. In summary, SSEA-4 marks potential MSC-like progenitor cells residing in the middle of porcine aortic valves.

We then examined co-expression of these different markers on pVICs and found that OB–CDH, NG2 and SSEA-4 labeled distinct cell subpopulations relative to each other, whereas ABCG2 and NG2 were co-expressed by the same cell subpopulation (Figure 2). ABCG2 is a well established marker for side population (SP) stem cells [19,37]. SP stem cells or progenitor cells are present in multiple tissues, including bone marrow, heart, skeletal muscle, liver, and mammary glands, where they can mediate tissue homeostasis and repair [19]. These cells are characterized by the efflux of the DNA dye Hoechst 33342, which is mainly determined by the expression of ABCG2, a multidrug resistance protein [19]. These SP cells have the stem cell properties of self-renewal, proliferation, and differentiation into the relevant lineages in the tissues where they reside. Recently, it has been shown that ABCG2 is not just a cell surface transporter on SP stem cells but also regulates the G1-to-S phase transition and determines symmetric vs. asymmetric division of cardiac SP progenitor cells [38]. Pho et al. have shown that ~0.5% of aortic pVICs have the SP cell property of dye efflux and can differentiate into myofibroblasts in response to serum treatment [13]. Consistently, we observe that pVICs from both aortic and pulmonary valves express the SP cell marker, ABCG2, but at a much higher percentage of ~5% (Table 2). This difference in percentage could be due to the different methods utilized (i.e., a dye efflux assay in the Pho et al. paper and the antibody staining in this study). The ABCG2^+^ cells are mostly positive for NG2, and NG2 is a cell-surface proteoglycan highly expressed by mural cells or pericytes, a type of progenitor closely associated with microvascular tubes during development [39]. NG2 is also expressed by oligodendrocyte progenitors in the central nervous system [20]. Our data suggest that these ABCG2^+^ cells may be progenitors in the valves and are consistent with the observation that ABCG2^+^ cells in the interstitium of skeletal muscles also express NG2 [40]. In that study, lineage tracing was performed and showed that, upon skeletal muscle injury, the progeny of ABCG2^+^ cells contributed primarily to endothelial cells and minimally to regenerated muscle fibers [40]. It would be worthwhile to carry out lineage tracing experiments in cardiac valves and examine the contribution of ABCG2^+^ cells in valvular development and disease.

The ABCG2^+^ cells were further characterized based on CD31 expression (Figure 3C). Interestingly, a majority (>60%) of the ABCG2^+^ cells are CD31^-^, supporting that these cells are from the interstitial cells of the valves. However, there are cells that express both ABCG2 and CD31, indicating that at least a portion of these cells have an endothelial origin. As ABCG2 and NG2 co-localize in the same cell subpopulation, NG2 staining should reflect ABCG2 staining. When the valve sections were stained with the NG2 antibody (as the ABCG2 antibody did not work for immunohistochemistry), cells along the edge of the valves were stained positive, indicating that NG2 labeled some endothelial or sub-endothelial cells (Figure 4C). However, we did not observe NG2 staining in the body of the leaflets. It is possible that the intra-valvular antigen is not exposed properly for the antibodies to bind to, so this result needs to be further confirmed using an ABCG2 antibody that will work for immunohistochemistry. Meanwhile, it has been shown that the endothelium of cardiac valves contains progenitors which not only play a critical role in initial cardiac cushion formation, but also maintain their differentiation potential in adult valves and may contribute to the repair of valvular injuries [41–43]. Our results indicate that 30-40% of ABCG2^+^ valvular cells express CD31 (Figure 3C), and these cell surface markers (ABCG2 and NG2) might be utilized in future research to isolate valvular endothelial subpopulations.

To further characterize the ABCG2^+^ cells, we efficiently sorted these cells and expanded them to a large number for *in vitro* differentiation assays. Expression of osteogenic genes in VIC populations has been observed in diseased valves [9], so osteogenic differentiation presents a relevant cell fate to study in pVICs [9,11,12]. Interestingly, when cultured under osteogenic conditions, ABCG2^+^ progeny produced a larger area of calcium mineralization than ABCG2^-^ progeny by day 8 (Figure 5B). In support of the existence of a progenitor cell population in valves, the Simmons group observed that pVICs as a heterogeous population could differentiate into three mesenchymal lineages, including osteoblasts, adipocytes and chondrocytes [12]. Our data is consistent with this work in showing the osteogenic potential of the ABCG2^+^ cells, but also implies that there could be different subpopulations present in pVICs which have different capacities of differentiation. For example, it is observed that ABCG2^+^ and NG2^+^ cells are present on both sides of aortic valves; however, the fibrosa side of the valves is more susceptible to calcification [44,45]. We hypothesize that this may be due to excessive collagen deposition and high stresses that may be present in the fibrosa side of valves, which could lead to more tissue damage and the recruitment of inflammatory cells to this site. In response to pathogenic factors present in the fibrosa, including increased matrix stiffness and chemical factors (such as TGF-β1), local ABCG2^+^ and NG2^+^ cells are more likely to be activated to participate in tissue repair or disease progression. Admittedly, even ABCG2^+^ cells are not a homogeneous population (Figure 3B). Additional molecular markers or clonally-derived cell cultures could be used to further define and compare this group of cells. Further, we observe that the ABCG2^+^ cells lose the ABCG2 expression after being cultured on plastic plates (Figure S2). This is not too surprising, as progenitor/stem cells are known to undergo spontaneous differentiation upon their non-physiological culture on plastic plates, and stem cell makers are easily lost during the process. However, the progeny of the ABCG2^+^ cells still had higher calcification capacities than the ABCG2^-^ cells, suggesting that progeny of the progenitor-like cells may still maintain different properties than the rest of the population. As progenitor/stem cells require appropriate chemical cues and physical cues for preserving their innate and primitive phenotypes [46,47], in the future, sorted ABCG2^+^ cells might be introduced to extracellular matrix-coated surfaces of defined elasticities, closer to valve tissue in stiffness, to enhance cell survival and maintain a more native-like phenotype [48–50]. Our study here presents an early effort in dissecting the complexity of cells residing in porcine aortic valves and provides evidence for heterogeneity not only in cellular composition, but also in molecular functions.

**Figure 6:**
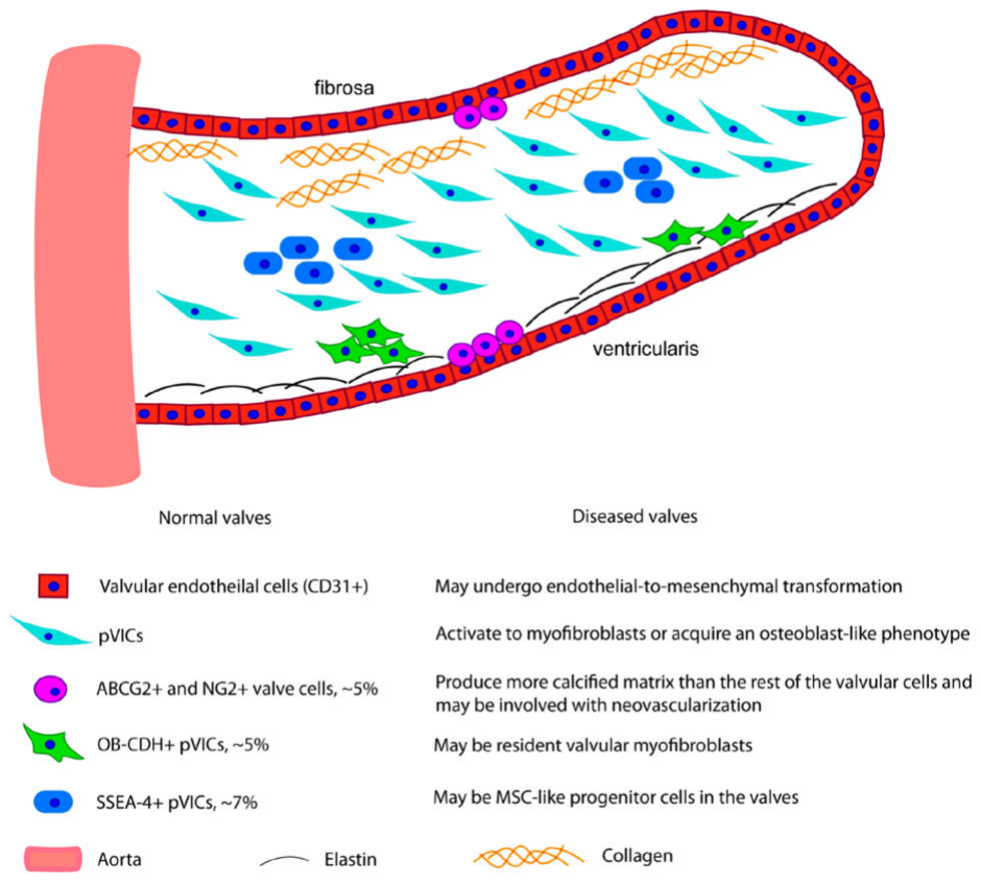
Porcine aortic valves are composed of different cell subpopulations. Based on previous literature and our flow cytometry data, porcine aortic valves are comprised of layers of endothelial cells and VICs. pVICs are further divided into multiple different subpopulations, including ABCG2^+^ and NG2^+^ pVICs, OB–CDH^+^ pVICs and SSEA-4^+^ pVICs. The functions of these different subpopulations in the diseased valves are speculated based on literature and our experimental data, and these serve as hypotheses to be tested in future.

In summary, porcine cardiac valves harbor different cell subpopulations which can be defined based on molecular markers. As shown in Figure 6, cardiac valves are composed of CD31^+^ endothelial cells as an outer layer and mostly fibroblasts, or pVICs, in the body of the leaflets. We have discovered that a subpopulation of pVICs expresses both ABCG2 and NG2, and produce more calcified matrix than the rest of the cells in osteogenic conditions. In addition, there are also OB–CDH^+^ pVICs, which may be resident myofibroblasts, in the valve. A small percentage of pVICs express SSEA-4, a MSC marker, and is postulated to be MSC-like progenitors residing near the middle of the valve. It is worthwhile to study the cellular functions of these cells individually or coordinately to understand the interplay between the different cellular subpopulations and how they mediate valve homeostasis and disease progression in a coordinated fashion.

## Conclusion

Given the increasing prevalence of valvular diseases and the role that VICs play in mediating valve repair and disease progression, these cells need to be defined not only based on their location, but also based on molecular markers. In this study, we characterized freshly isolated pVICs based on lineage-associated protein makers (CD31), as well as other important markers either associated with myofibroblast differentiation (e.g., OB–CDH), or related to progenitor subpopulations (e.g., ABCG2, NG2, SSEA4). Flow cytometry results demonstrate that small fractions of pVICs express OB–CDH (~5%), ABCG2 (~5%), NG2 (~5%) or SSEA-4 (7-14%), and that pVICs isolated from either aortic or pulmonary valves express similar levels of these cell surface markers, except SSEA-4. Significantly more pVICs express SSEA-4 in pulmonary valves than in aortic valves. Interestingly, OB–CDH, NG2 and SSEA-4 all label distinct pVIC subpopulations relative to each other; however, ABCG2 and NG2 are co-expressed by the same cell subpopulation.

We have further examined the ABCG2^+^ cells as a model for studying molecular functions of a specified valvular subpopulation. The ABCG2^+^ cells are from both pVICs and endothelial cells, and deposit more calcium than the rest of the population under osteogenic culture conditions, indicating that this population may play a more prominent role in the process of valve calcification. In the future, each specific VIC subpopulation could be sorted based on these markers and studied for their functional properties, which should lead to a better understanding as to how VICs, as a mixed population, maintain valvular homeostasis and participate in tissue repair and pathology.

## Supporting Information

Figure S1 Efficiency of the Collagenase II digestion. pVICs were isolated from aortic valves based on a standard method as described in the Materials and Methods. After the second Collagenase II digestion, the leftover tissues were subjected to another 20 hours of digestion and 2 min vortex, which completely dissolved the tissues. Based on the total cell number released at each step, we found that 78.3 ± 7.9% of pVICs were released after the second Collagenase II digestion, and 21.7 ± 7.9% of pVICs were released after complete collagenase digestion.
(TIF)

Figure S2 Expression of the stem cell marker ABCG2 is lost after plastic plate culture. We examined whether expression of ABCG2 was maintained during VIC culture on plastic plates. In the figure, the y-axis represents the fluorescence intensity of ABCG2 staining and the x-axis is forward scattering. Gates were set based on the isotype control staining. (A) ABCG2^+^ cells (Gate R1) and ABCG2^-^ cells (Gate R2) were sorted at equal amounts based on positive or negative staining of ABCG2. (B) After ~2 weeks of propagation on plastic plates, sorted ABCG2^+^ valvular cells lost the expression of ABCG2 based on flow cytometry.
(TIF)

## References

[1] Mohler ER III (2004) Mechanisms of aortic valve calcification. Am J Cardiol 94: 1396-1402. doi:10.1016/j.amjcard.2004.08.013. PubMed: 15566910. 15566910 10.1016/j.amjcard.2004.08.013

[2] Rabkin-Aikawa E [!(surname)!], Aikawa M, Schoen FJ (2004) Dynamic and Reversible Changes of Interstitial Cell Phenotype During Remodeling of Cardiac Valves. J Heart Valve Dis 13: 841-847. PubMed: 15473488. 15473488

[3] Aikawa E, Whittaker P, Farber M, Mendelson K, Padera RF et al. (2006) Human semilunar cardiac valve remodeling by activated cells from fetus to adult: Implications for postnatal adaptation, pathology, and tissue engineering. Circulation 113: 1344-1352. doi:10.1161/CIRCULATIONAHA.105.591768. PubMed: 16534030. 16534030 10.1161/CIRCULATIONAHA.105.591768

[4] Hinton RB, Lincoln J, Deutsch GH, Osinska H, Manning PB et al. (2006) Extracellular Matrix Remodeling and Organization in Developing and Diseased Aortic Valves. Circ Res 98: 1431-1438. doi:10.1161/01.RES.0000224114.65109.4e. PubMed: 16645142. 16645142 10.1161/01.RES.0000224114.65109.4e

[5] Latif N, Sarathchandra P, Thomas PS, Antoniw J, Batten P et al. (2007) Characterization of Structural and Signaling Molecules by Human Valve Interstitial Cells and Comparison to Human Mesenchymal Stem Cells. J Heart Valve Dis 16: 56-66. PubMed: 17315384. 17315384

[6] Gould ST, Darling NJ, Anseth KS (2012) Small peptide functionalized thiol-ene hydrogels as culture substrates for understanding valvular interstitial cell activation and de novo tissue deposition. Acta Biomater 8: 3201-3209. doi:10.1016/j.actbio.2012.05.009. PubMed: 22609448. 22609448 10.1016/j.actbio.2012.05.009 PMC3470806

[7] Hinton RB, Yutzey KE (2011) Heart valve structure and function in development and disease. Annu Rev Physiol 73: 29-46. doi:10.1146/annurev-physiol-012110-142145. PubMed: 20809794. 20809794 10.1146/annurev-physiol-012110-142145 PMC4209403

[8] Rabkin E, Aikawa M, Stone JR, Fukumoto Y, Libby P et al. (2001) Activated Interstitial Myofibroblasts Express Catabolic Enzymes and Mediate Matrix Remodeling in Myxomatous Heart Valves. Circulation 104: 2525-2532. doi:10.1161/hc4601.099489. PubMed: 11714645. 11714645 10.1161/hc4601.099489

[9] Rajamannan NM, Subramaniam M, Rickard D, Stock SR, Donovan J et al. (2003) Human Aortic Valve Calcification Is Associated With an Osteoblast Phenotype. Circulation 107: 2181-2184. doi:10.1161/01.CIR.0000070591.21548.69. PubMed: 12719282. 12719282 10.1161/01.CIR.0000070591.21548.69 PMC3922288

[10] Liu AC, Joag VR, Gotlieb AI (2007) The Emerging Role of Valve Interstitial Cell Phenotypes in Regulating Heart Valve Pathobiology. Am J Pathol 171: 1407-1418. doi:10.2353/ajpath.2007.070251. PubMed: 17823281. 17823281 10.2353/ajpath.2007.070251 PMC2043503

[11] Rattazzi M, Iop L, Faggin E, Bertacco E, Zoppellaro G et al. (2008) Clones of Interstitial Cells From Bovine Aortic Valve Exhibit Different Calcifying Potential When Exposed to Endotoxin and Phosphate. Arterioscler Thromb Vasc Biol 28: 2165-2172. doi:10.1161/ATVBAHA.108.174342. PubMed: 18832754. 18832754 10.1161/ATVBAHA.108.174342

[12] Chen JH, Yip CY, Sone ED, Simmons CA (2009) Identification and Characterization of Aortic Valve Mesenchymal Progenitor Cells with Robust Osteogenic Calcification Potential. Am J Pathol 174: 1109-1119. doi:10.2353/ajpath.2009.080750. PubMed: 19218344. 19218344 10.2353/ajpath.2009.080750 PMC2665769

[13] Pho M, Lee W, Watt DR, Laschinger C, Simmons CA et al. (2008) Cofilin is a marker of myofibroblast differentiation in cells from porcine aortic cardiac valves. Am J Physiol Heart Circ Physiol 294: H1767-H1778. doi:10.1152/ajpheart.01305.2007. PubMed: 18263709. 18263709 10.1152/ajpheart.01305.2007

[14] Veinot JP, Prichett-Pejic W, Song J, Waghray G, Parks W et al. (2006) CD117-positive cells and mast cells in adult human cardiac valves--observations and implications for the creation of bioengineered grafts. Cardiovasc Pathol 15: 36-40. doi:10.1016/j.carpath.2005.08.005. PubMed: 16414455. 16414455 10.1016/j.carpath.2005.08.005

[15] Hajdu Z, Romeo SJ, Fleming PA, Markwald RR, Visconti RP, Drake CJ (2011) Recruitment of bone marrow-derived valve interstitial cells is a normal homeostatic process. J Mol Cell Cardiol 51: 955-965. doi:10.1016/j.yjmcc.2011.08.006. PubMed: 21871458. 21871458 10.1016/j.yjmcc.2011.08.006 PMC3208774

[16] Deb A, Wang SH, Skelding K, Miller D, Simper D et al. (2005) Bone marrow-derived myofibroblasts are present in adult human heart valves. J Heart Valve Dis 14: 674-678. PubMed: 16245507. 16245507

[17] Pusztaszeri MP, Seelentag W, Bosman FT (2006) Immunohistochemical Expression of Endothelial Markers CD31, CD34, von Willebrand Factor, and Fli-1 in Normal Human Tissues. J Histochem Cytochem 54: 385-395. doi:10.1369/jhc.4A6514.2005. PubMed: 16234507. 16234507 10.1369/jhc.4A6514.2005

[18] Hinz B, Pittet P, Smith-Clerc J, Chaponnier C, Meister JJ (2004) Myofibroblast Development Is Characterized by Specific Cell-Cell Adherens Junctions. Mol Biol Cell 15: 4310-4320. doi:10.1091/mbc.E04-05-0386. PubMed: 15240821. 15240821 10.1091/mbc.E04-05-0386 PMC515361

[19] Zhou S, Schuetz JD, Bunting KD, Colapietro AM, Sampath J et al. (2001) The ABC transporter Bcrp1/ABCG2 is expressed in a wide variety of stem cells and is a molecular determinant of the side-population phenotype. Nat Med 7: 1028-1034. doi:10.1038/nm0901-1028. PubMed: 11533706. 11533706 10.1038/nm0901-1028

[20] Stallcup WB (2002) The NG2 proteoglycan: Past insights and future prospects. J Neurocytol 31: 423-435. doi:10.1023/A:1025731428581. PubMed: 14501214. 14501214 10.1023/a:1025731428581

[21] Initiative TISC (2007) Characterization of human embryonic stem cell lines by the International Stem Cell Initiative. Nat Biotech 25: 803-816

[22] Gang EJ, Bosnakovski D, Figueiredo CA, Visser JW, Perlingeiro RC (2007) SSEA-4 identifies mesenchymal stem cells from bone marrow. Blood 109: 1743-1751. doi:10.1182/blood-2005-11-010504. PubMed: 17062733. 17062733 10.1182/blood-2005-11-010504

[23] Johnson CM, Hanson MN, Helgeson SC (1987) Porcine cardiac valvular subendothelial cells in culture: Cell isolation and growth characteristics. J Mol Cell Cardiol 19: 1185-1193. doi:10.1016/S0022-2828(87)80529-1. PubMed: 3327949. 3327949 10.1016/s0022-2828(87)80529-1

[24] Lee DM, Kiener HP, Agarwal SK, Noss EH, Watts GF et al. (2007) Cadherin-11 in Synovial Lining Formation and Pathology in Arthritis. Science 315: 1006-1010. doi:10.1126/science.1137306. PubMed: 17255475. 17255475 10.1126/science.1137306

[25] Benoit DS, Nuttelman CR, Collins SD, Anseth KS (2006) Synthesis and characterization of a fluvastatin-releasing hydrogel delivery system to modulate hMSC differentiation and function for bone regeneration. Biomaterials 27: 6102-6110. doi:10.1016/j.biomaterials.2006.06.031. PubMed: 16860387. 16860387 10.1016/j.biomaterials.2006.06.031

[26] Pittenger MF, Mackay AM, Beck SC, Jaiswal RK, Douglas R et al. (1999) Multilineage Potential of Adult Human Mesenchymal Stem Cells. Science 284: 143-147. doi:10.1126/science.284.5411.143. PubMed: 10102814. 10102814 10.1126/science.284.5411.143

[27] Paranya G, Vineberg S, Dvorin E, Kaushal S, Roth SJ et al. (2001) Aortic Valve Endothelial Cells Undergo Transforming Growth Factor-beta-Mediated and Non-Transforming Growth Factor-beta-Mediated Transdifferentiation in Vitro. Am J Pathol 159: 1335-1343. doi:10.1016/S0002-9440(10)62520-5. PubMed: 11583961. 11583961 10.1016/s0002-9440(10)62520-5 PMC1850524

[28] Müller AM, Hermanns MI, Skrzynski C, Nesslinger M, Müller KM et al. (2002) Expression of the Endothelial Markers PECAM-1, vWf, and CD34 in Vivo and in Vitro. Exp Mol Pathol 72: 221-229. doi:10.1006/exmp.2002.2424. PubMed: 12009786. 12009786 10.1006/exmp.2002.2424

[29] Pittet P, Lee K, Kulik AJ, Meister JJ, Hinz B (2008) Fibrogenic fibroblasts increase intercellular adhesion strength by reinforcing individual OB-cadherin bonds. J Cell Sci 121: 877-886. doi:10.1242/jcs.024877. PubMed: 18303045. 18303045 10.1242/jcs.024877

[30] Walker GA, Masters KS, Shah DN, Anseth KS, Leinwand LA (2004) Valvular myofibroblast activation by transforming growth factor-beta: Implications for pathological extracellular matrix remodeling in heart valve disease. Circ Res 95: 253-260. doi:10.1161/01.RES.0000136520.07995.aa. PubMed: 15217906. 15217906 10.1161/01.RES.0000136520.07995.aa

[31] Chen JH, Chen WL, Sider KL, Yip CY, Simmons CA (2011) β-catenin mediates mechanically regulated, transforming growth factor-β1-induced myofibroblast differentiation of aortic valve interstitial cells. Arterioscler Thromb Vasc Biol 31: 590-597. doi:10.1161/ATVBAHA.110.220061. PubMed: 21127288. 21127288 10.1161/ATVBAHA.110.220061

[32] Cushing MC, Mariner PD, Liao JT, Sims EA, Anseth KS (2008) Fibroblast growth factor represses Smad-mediated myofibroblast activation in aortic valvular interstitial cells. FASEB J 22: 1769-1777. doi:10.1096/fj.07-087627. PubMed: 18218921. 18218921 10.1096/fj.07-087627 PMC2493079

[33] Kii I, Amizuka N, Shimomura J, Saga Y, Kudo A (2004) Cell-Cell Interaction Mediated by Cadherin-11 Directly Regulates the Differentiation of Mesenchymal Cells Into the Cells of the Osteo-Lineage and the Chondro-Lineage. Journal of Bone and Mineral Research 19: 1840-1849. 15476585 10.1359/JBMR.040812

[34] Okazaki M, Takeshita S, Kawai S, Kikuno R, Tsujimura A et al. (1994) Molecular cloning and characterization of OB-cadherin, a new member of cadherin family expressed in osteoblasts. J Biol Chem 269: 12092-12098. PubMed: 8163513. 8163513

[35] Di Benedetto A, Watkins M, Grimston S, Salazar V, Donsante C et al. (2010) N-cadherin and cadherin 11 modulate postnatal bone growth and osteoblast differentiation by distinct mechanisms. J Cell Sci 123: 2640-2648. doi:10.1242/jcs.067777. PubMed: 20605916. 20605916 10.1242/jcs.067777 PMC2908051

[36] Simonneau L, Kitagawa M, Suzuki S, Thiery JP (1995) Cadherin 11 Expression Marks the Mesenchymal Phenotype: Towards New Functions for Cadherins? Cell Adhes Commun 3: 115-130. doi:10.3109/15419069509081281. PubMed: 7583005. 7583005 10.3109/15419069509081281

[37] Ding XW, Wu JH, Jiang CP (2010) ABCG2: A potential marker of stem cells and novel target in stem cell and cancer therapy. Life Sci 86: 631-637. doi:10.1016/j.lfs.2010.02.012. PubMed: 20159023. 20159023 10.1016/j.lfs.2010.02.012

[38] Sereti KI, Oikonomopoulos A, Unno K, Cao X, Qiu Y et al. (2013) ATP-Binding Cassette G-Subfamily Transporter 2 Regulates Cell Cycle Progression and Asymmetric Division in Mouse Cardiac Side Population Progenitor Cells. Circ Res 112: 27-34. doi:10.1161/CIRCRESAHA.111.300010. PubMed: 23136123. 23136123 10.1161/CIRCRESAHA.111.300010 PMC3959170

[39] Ozerdem U, Grako KA, Dahlin-Huppe K, Monosov E, Stallcup WB (2001) NG2 proteoglycan is expressed exclusively by mural cells during vascular morphogenesis. Dev Dyn 222: 218-227. doi:10.1002/dvdy.1200. PubMed: 11668599. 11668599 10.1002/dvdy.1200

[40] Doyle MJ, Zhou S, Tanaka KK, Pisconti A, Farina NH et al. (2011) Abcg2 labels multiple cell types in skeletal muscle and participates in muscle regeneration. J Cell Biol 195: 147-163. doi:10.1083/jcb.201103159. PubMed: 21949413. 21949413 10.1083/jcb.201103159 PMC3187700

[41] Wylie-Sears J, Aikawa E, Levine RA, Yang JH, Bischoff J (2011) Mitral Valve Endothelial Cells With Osteogenic Differentiation Potential. Arterioscler Thromb Vasc Biol 31: 598-607. doi:10.1161/ATVBAHA.110.216184. PubMed: 21164078. 21164078 10.1161/ATVBAHA.110.216184 PMC3210435

[42] Bischoff J, Aikawa E (2011) Progenitor Cells Confer Plasticity to Cardiac Valve Endothelium. J Cardiovasc Transl Res 4: 710-719. doi:10.1007/s12265-011-9312-0. PubMed: 21789724. 21789724 10.1007/s12265-011-9312-0

[43] Paruchuri S, Yang JH, Aikawa E, Melero-Martin JM, Khan ZA et al. (2006) Human Pulmonary Valve Progenitor Cells Exhibit Endothelial/Mesenchymal Plasticity in Response to Vascular Endothelial Growth Factor-A and Transforming Growth Factor-beta2. Circ Res 99: 861-869. doi:10.1161/01.RES.0000245188.41002.2c. PubMed: 16973908. 16973908 10.1161/01.RES.0000245188.41002.2c PMC2810464

[44] O’Brien KD, Reichenbach DD, Marcovina SM, Kuusisto J, Alpers CE et al. (1996) Apolipoproteins B, (a), and E Accumulate in the Morphologically Early Lesion of 'Degenerative' Valvular Aortic Stenosis. Arterioscler Thromb Vasc Biol 16: 523-532. doi:10.1161/01.ATV.16.4.523. PubMed: 8624774. 8624774 10.1161/01.atv.16.4.523

[45] Chen JH, Simmons CA (2011) Cell-Matrix Interactions in the Pathobiology of Calcific Aortic Valve Disease: Critical Roles for Matricellular, Matricrine, and Matrix Mechanics Cues. Circ Res 108: 1510-1524. doi:10.1161/CIRCRESAHA.110.234237. PubMed: 21659654. 21659654 10.1161/CIRCRESAHA.110.234237

[46] Zapata AG, Alfaro D, García-Ceca J, López-Larrea C, López-Vázquez A et al. (2012) Biology of Stem Cells: The Role of Microenvironments. Stem Cell Transplantation: Advances Experimental Med Biol, 741: 135-151. PubMed: 22457108. 10.1007/978-1-4614-2098-9_10 22457108

[47] Wagers AJ (2012) The Stem Cell Niche in Regenerative Medicine. Cell Stem Cell 10: 362-369. doi:10.1016/j.stem.2012.02.018. PubMed: 22482502. 22482502 10.1016/j.stem.2012.02.018

[48] Wang H, Haeger SM, Kloxin AM, Leinwand LA, Anseth KS (2012) Redirecting Valvular Myofibroblasts into Dormant Fibroblasts through Light-mediated Reduction in Substrate Modulus. PLOS ONE 7: e39969. doi:10.1371/journal.pone.0039969. PubMed: 22808079. 22808079 10.1371/journal.pone.0039969 PMC3396623

[49] Gilbert PM, Blau HM (2011) Engineering a stem cell house into a home. Stem Cell Res Ther 2: 3. doi:10.1186/scrt44. PubMed: 21345268. 21345268 10.1186/scrt44 PMC3092143

[50] Kloxin AM, Kasko AM, Salinas CN, Anseth KS (2009) Photodegradable Hydrogels for Dynamic Tuning of Physical and Chemical Properties. Science 324: 59-63. doi:10.1126/science.1169494. PubMed: 19342581. 19342581 10.1126/science.1169494 PMC2756032

[51] Martin CM, Meeson AP, Robertson SM, Hawke TJ, Richardson JA et al. (2004) Persistent expression of the ATP-binding cassette transporter, Abcg2, identifies cardiac SP cells in the developing and adult heart. Dev Biol 265: 262-275. doi:10.1016/j.ydbio.2003.09.028. PubMed: 14697368. 14697368 10.1016/j.ydbio.2003.09.028

