# Supplementary figures and images for: Characterization of Cell Subpopulations Expressing Progenitor Cell Markers in Porcine Cardiac Valves
Source: PLoS One. 2013 Jul 23;8(7):e69667. doi: 10.1371/journal.pone.0069667 (PMC3720586; doi:10.1371/journal.pone.0069667)

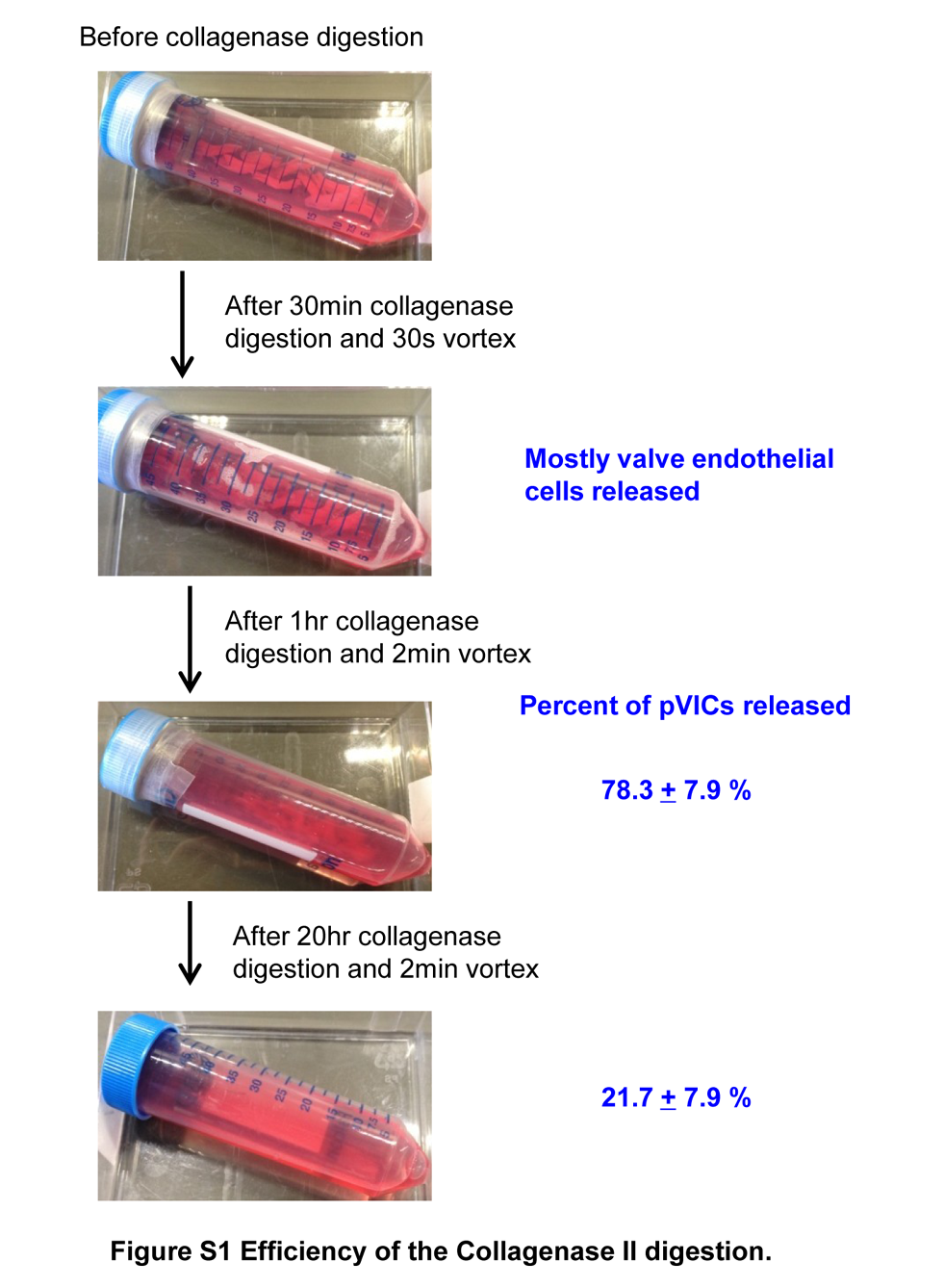

Supplement: Figure S1 — pVICs were isolated from aortic valves based on a standard method as described in the Materials and Methods. After the second Collagenase II digestion, the leftover tissues were subjected to another 20 hours of digestion and 2 min vortex, which completely dissolved the tissues. Based on the total cell number released at each step, we found that 78.3 ± 7.9% of pVICs were released after the second Collagenase II digestion, and 21.7 ± 7.9% of pVICs were released after complete collagenase digestion. (TIF) [file pone.0069667.s001.tif]

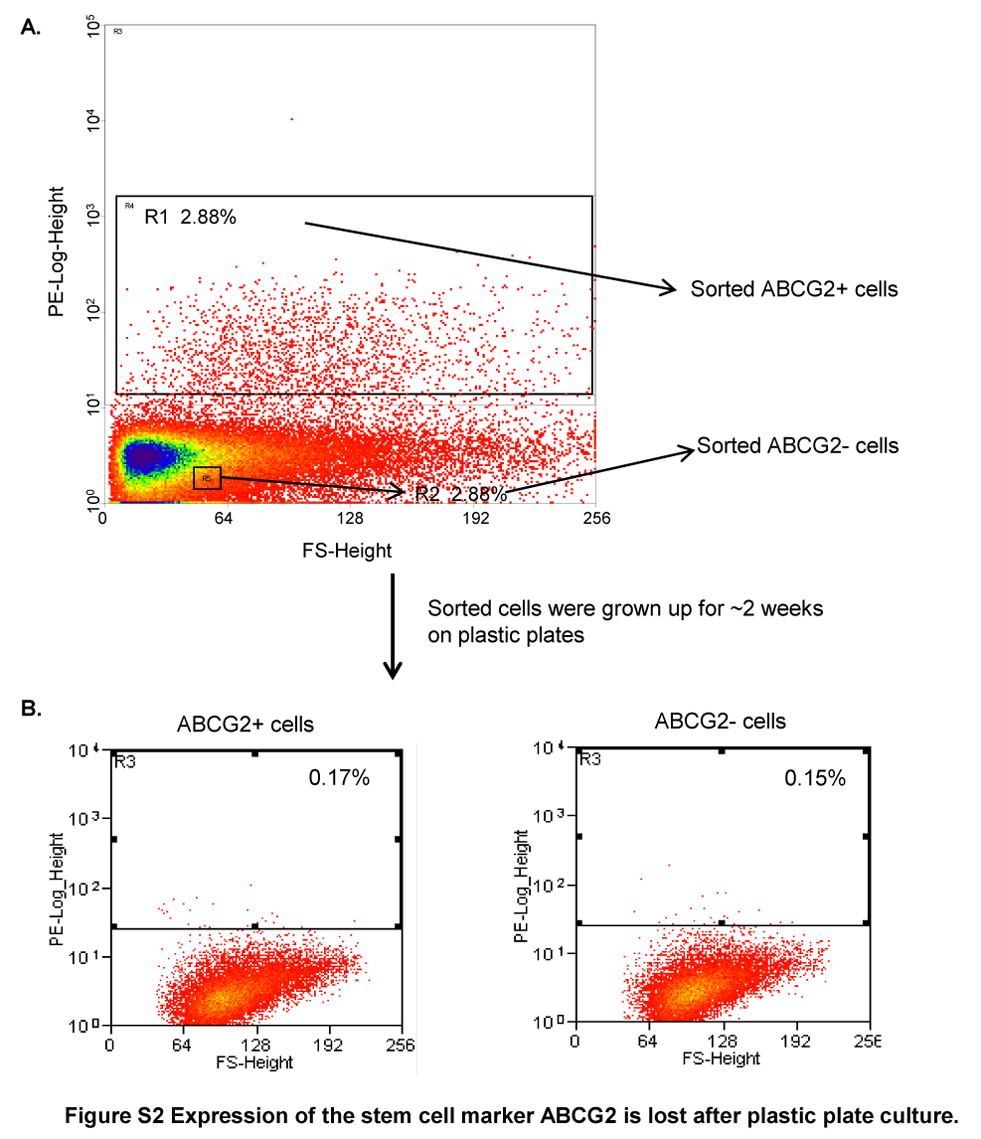

Supplement: Figure S2 — We examined whether expression of ABCG2 was maintained during VIC culture on plastic plates. In the figure, the y-axis represents the fluorescence intensity of ABCG2 staining and the x-axis is forward scattering. Gates were set based on the isotype control staining. (A) ABCG2+ cells (Gate R1) and ABCG2- cells (Gate R2) were sorted at equal amounts based on positive or negative staining of ABCG2. (B) After ~2 weeks of propagation on plastic plates, sorted ABCG2+ valvular cells lost the expression of ABCG2 based on flow cytometry. (TIF) [file pone.0069667.s002.tif]
